# The long-term effect of intentional weight loss on changes in bone mineral density in persons with type 2 diabetes: results from the Look AHEAD randomized trial

**DOI:** 10.1007/s11657-023-01303-0

**Published:** 2023-07-14

**Authors:** Karen C. Johnson, Andrea Anderson, Kristen M. Beavers, Carolyn J. Crandall, Helen P. Hazuda, Cora E. Lewis, Edward Lipkin, Ann V. Schwartz, F. X. Pi-Sunyer, Qi Zhao, and the Look AHEAD Research Group

**Affiliations:** 1 Department of Preventive Medicine, University of Tennessee Health Science Center, Memphis, TN USA; 2 Wake Forest Baptist Health, Winston-Salem, NC USA; 3 Department of Health and Exercise Science, Wake Forest Univesity, Winston-Salem, NC USA; 4 Department of Medicine, David Geffen School of Medicine at University of California, Los Angeles, CA USA; 5 Univesity of Texas Health Science Center at San Antonio, San Antonio, TX USA; 6 Depatment of Epidemiology, Univeristy of Alabama at Birmingham, Birmingham, AL USA; 7 Division of Metabolism, Endocrinology and Nutrition, University of Washington, Seattle, WA USA; 8 Deparment of Epidemiology and Biostatistics, University of California, San Francisco, CA USA; 9 Department of Medicine, Columbia University, New York, NY USA

**Keywords:** BMD, Intentional weight loss, Type 2 diabetes, Clinical trial

## Abstract

***Summary*:**

Intentional weight loss has been shown to increase bone loss short term but the long-term effects are not known. Data from the Look AHEAD clinical trial shows that a long term intentional weight loss intervention was associated with greater bone loss at the hip in men.

**Purpose:**

Intentional weight loss has been shown to increase bone loss short term and increase frailty fracture risk, but the long-term effects on bone mineral density (BMD) are not known.

**Methods:**

Data from a subgroup from the Look AHEAD (LA) multicenter, randomized clinical trial was used to evaluate whether a long term intentional weight loss intervention would increase bone loss. In a preplanned substudy, BMD was assessed at 5 of the 16 LA clinical centers using dual-energy X-ray absorptiometry at baseline, year 8, and the observational visit 12.6–16.3 years after randomization (year 12–16).

**Results:**

At year 8, bone density loss (%) was greater in the Intensive Lifestyle Intervention (ILI) group compared with the control group (DSE) for the femoral neck (*p* = 0.0122) but this finding was not observed at the year 12–16 visit. In analyses stratified by gender, bone density loss (%) was greater at the total hip for men in the ILI group than the DSE group at both the year 8 and year 12–16 visits (year 8 *p* = 0.0263 and year 12–16 *p* = 0.0062). This finding was not observed among women.

**Conclusion:**

Long term intentional weight loss was associated with greater bone loss at the hip in men. These results taken with the previously published Look AHEAD data from the entire clinical trial showing increased frailty fracture risk with weight loss in the ILI group suggest that when intentional weight loss is planned, consideration of bone density preservation and fracture prevention strategies is warranted.

**Trial Registration:**

Clinicaltrials.gov Identifier: NCT00017953. June 21, 2001

**Supplementary Information:**

The online version contains supplementary material available at 10.1007/s11657-023-01303-0.

## Introduction

Among the US population, it is estimated that over 34 million people have diabetes and this risk increases with age and increasing body mass index (BMI) [1]. Weight loss is often recommended for persons with diabetes to improve glycemic control [2]. Unfortunately, in the Look AHEAD clinical trial in which an intensive lifestyle intervention (ILI) of decreased caloric consumption and increased physical activity was compared to a comparison condition of diabetes support and education (DSE), intentional weight loss increased bone loss short term (1 to 4 years) in the DXA subgroup and increased frailty fracture risk in the whole Look AHEAD clinical trial in the ILI group compared to DSE in persons with type 2 diabetes (DM) [3–5]. However, the net effect of intentional weight loss over an extended period of time on bone mineral density in older persons with diabetes is not known.

Therefore, the purpose of this analysis is to examine whether this long term intentional weight loss intervention resulted in sustained bone density loss, beyond four years, as measured by dual-energy X-ray absorptiometry (DXA) in older persons with overweight / obesity living with type 2 DM in the Look AHEAD randomized clinical trial.

## Methods

The Look AHEAD randomized clinical trial involved 16 clinical sites across the US (Clinicaltrials.gov Identifier: NCT00017953). The methods and baseline characteristics of the study population have been published and the protocol is available (https://repository.niddk.nih.gov/studies/look-ahead/). [6, 7] The primary goal of Look AHEAD was to determine whether randomization (1:1 allocation ratio) to ILI reduced cardiovascular morbidity and mortality relative to DSE among individuals with overweight or obesity and with type 2 DM. On September 14, 2012, the clinical trial intervention was stopped for futility because there was no difference with regard to the primary cardiovascular endpoints between ILI and DSE [8]. At that time, all participants had completed 8 to 12 years of the intervention. After 2012, Look AHEAD has continued as a prospective cohort. All Look AHEAD participants alive at the end of the trial when the intervention was stopped were invited to join a follow-up observational study to determine the longer term effects of the intervention on a number of outcomes. This paper reports on change in areal bone mineral density (BMD) data obtained from DXA scans from baseline (2001–2004) through April 2018.

Intervention curricula for both ILI and DSE were developed centrally and have been described in detail [9]. ILI aimed at achieving and maintaining at least a 7% weight loss by focusing on reduced caloric intake and increased physical activity. The program included frequent contact throughout the trial, with both group and individual sessions, a calorie goal of 1200–1800 kcal/day (< 30% of calories from fat and > 15% from protein), use of meal replacement products, and at least 175 min per week of moderate intensity physical activity. The most common type of physical activity was walking. A toolbox of strategies was available for participants having difficulty achieving the weight loss goals. The intervention did not include any advice regarding prevention of bone loss during weight loss.

Look AHEAD was approved by local Institutional Review Boards and all participants provided informed consent. Look AHEAD complied with the World Medical Association Declaration of Helsinki – Ethical Principles for Medical Research Involving Human Subjects.

Major eligibility criteria for Look AHEAD included the following: 45–76 years of age; type 2 DM; body mass index (BMI) of ≥ 25.0 kg/m^2^ (≥ 27 in individuals taking insulin); hemoglobin A1c (HbA1c) < 11%, systolic blood pressure (SBP) < 160 mmHg, diastolic blood pressure (DBP) < 100 mmHg, and triglycerides < 600 mg/dl; ability to complete a valid maximal exercise test suggesting it was safe to exercise; and having a primary care provider. Participants could be using any type of glucose-lowering medication, but the percentage of participants using insulin was limited to < 30%.

Randomization occurred from August 2001 through April 2004 with an allocation ratio of 1:1 and stratification by clinical site. At baseline and annual clinic visits, weight and height were measured with a digital scale and a standard wall-mounted stadiometer respectively. BMI was calculated from the measured weights and heights. Questionnaires were used to collect demographic characteristics, medical history, smoking history, and alcohol use. Fracture data was collected as previously described in the Look AHEAD fracture paper [5]. Race categories are self-reported. Blood work was completed after an overnight fast and was analyzed by the Central Biochemistry Laboratory (Northwest Lipid Research Laboratories, University of Washington, Seattle, WA) using standardized laboratory procedures. Participants were instructed to bring all prescription medicines to the clinic annually for a medication inventory; when participants did not bring their medications, staff determined if there were any changes from the previous visit, placing follow-up phone calls when necessary. Bone-active medications were classified from the medication inventory and are used in these analyses. Bone-negative medications were defined as: loop diuretics, selective serotonin reuptake inhibitors (SSRIs), thyroid hormones, oral glucocorticoids such as prednisone, tricyclic antidepressants, and thiazolidinedione (TZDs). Bone-positive medications were defined as: androgens (anabolic steroids), calcium, vitamin D, antacids containing calcium, and antiresorptive agents such as bisphosphonates, calcitonin nasal spray, estrogens, and selective estrogen receptor modulators (SERMs). During the DXA substudy, no other bone-positive medications were taken by the Look AHEAD participants even when they were available for use.

## Dual-energy X-ray absorptiometry (DXA) substudy

**Fig. 1:**
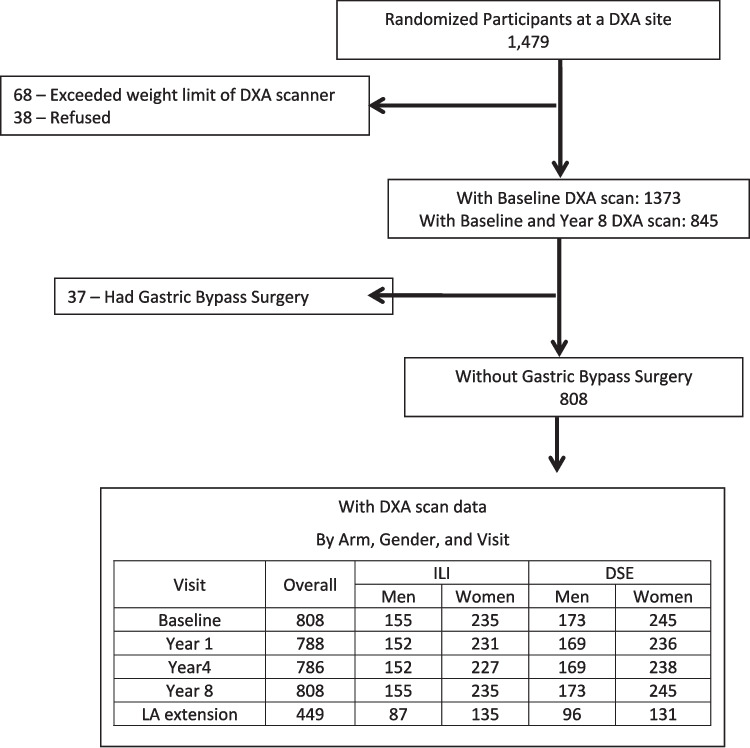
Consort diagram for the Look AHEAD DXA substudy participants

Look AHEAD was also designed to examine secondary outcomes including areal BMD changes. In a preplanned substudy, total hip, lumbar spine, and whole body DXA scans were obtained at baseline, year 1, year 4, year 8, and at the observational visit which occurred 12.6 – 16.3 years after randomization (year 12–16) at five clinical centers (Baton Rouge, Boston, Houston, Los Angeles, and Seattle). This paper extends the BMD findings previously published to the year 8 and year 12–16 visit [3, 4]. Sites were selected based on availability of DXA scanners and interest of the local investigators. Of 1479 enrolled randomized participants at the DXA sites, 68 were ineligible for the DXA substudy because their weight exceeded the DXA scanner limit (> 300 lbs.), 38 refused the DXA substudy leaving 1373 who had a baseline DXA scan of which 845 also had a DXA scan at year 8. An additional 37 persons were excluded from these analyses due to a gastric bypass surgery during the Look AHEAD trial leaving 808 included in these analyses (Fig. 1). All DXA sites used Hologic fan beam densitometers, and any software upgrades during the study were approved by the DXA quality assurance center (San Francisco Coordinating Center, University of California San Francisco). Longitudinal performance was monitored with regular scanning of a spine and a whole body phantom on each densitometer. The mineral content of bone was defined for an area of interest from an AP DXA image as grams (g) calcium per two dimensional area of bone in centimeters (cm) squared (areal BMD) and not as the true volumetric calcium content. The coefficient of variation for spine and whole-body phantom BMD ranged from 0.36% to 0.39% and 1.6% to 2.4%, respectively, at the five clinical sites. Longitudinal corrections were applied to spine and hip BMD results at the Los Angeles site and to whole body BMD at the Houston site. Soft tissue results were corrected to account for underestimation of fat mass [10]. The quality of participant scans was centrally monitored. T-scores were calculated using a young white female reference group, from the National Health and Nutrition Examination Survey (NHANES III) for total hip and the manufacturer’s database for spine as recommended by the International Society for Clinical Densitometry (ISCD). If BMD loss was > 10% for lumbar spine or total hip, the participant and primary care provider were notified, but participants were not asked to stop the study intervention.

## Fracture ascertainment

During annual visits and telephone calls every 6 months, staff members who were unaware of study-group assignments (blinded) queried participants about all medical events and hospitalizations including incident fractures. Hospital and other records such as outpatient medical records and x-ray reports were reviewed for potential incident fracture events, with adjudication according to standard criteria by a central review committee of trained physicians who were blinded to study-group assignment. The primary fracture outcome was prespecified as the first occurrence of a fracture. Self-reported fractures of the fingers, toes, face, neck (c-spine), sternum, ribs, and skull were not centrally adjudicated and are not included in the fracture events for this manuscript. Only confirmed centrally adjudicated incident fracture events are included in these analyses. As a secondary fracture outcome, a frailty fracture endpoint was created a priori and is a composite of the first occurrence of a hip, pelvis, upper arm or shoulder fracture [11]. This secondary fracture endpoint was selected because data from the Study of Osteoporotic Fractures (SOF) had previously demonstrated that weight loss was associated with frailty fracture [11].

## Statistical analysis

Baseline characteristics were summarized with means and standard deviations for continuous variables, and frequencies and percents for categorical variables, stratified by randomization arm and gender. Comparisons between randomization arms were made with t-tests or chi-square tests of association as appropriate with a p value of < 0.05 accepted as significant. Absolute changes in BMD were compared across ILI and DSE groups. Analyses are also presented stratified by gender because of well-described gender differences in BMD and bone loss. Differences by randomization assignment were tested with longitudinal linear models to account for within-subject correlation. All models are adjusted for study site, age, race/ethnicity, baseline total bone mass, use of bone positive agents, use of bone negative agents, and baseline value of the BMD outcome. Overall models are also adjusted for gender. Gender differences in percent changes in BMD by visit are explored in overall models that also include an interaction term. Adjusted mean change from baseline and standard errors are reported. Analyses were carried out using SAS v 9.4 (SAS Institute, Cary, NC).

## Results

At baseline, the participants in the DXA substudy included in this manuscript were on average 59 years old, 59% were women, 74% were Caucasian, the mean BMI was 35 kg/m^2^, and the majority of women were postmenopausal (81%). Overall, there were no statistically significant baseline differences between the ILI and DSE groups including duration of diabetes, household income, years of education, smoking status, alcohol intake, physical activity, depressive symptoms, use of bone-active agents or insulin, estimated glomerular filtration rate (eGFR), or hemoglobin A1c (HbA1c). At baseline among women only, the ILI group was more physically active than the DSE group (*p* = 0.0286) and the ILI group had a lower HbA1c than the DSE group (*p* = 0.0187), however, no other significant differences were noted (Table 1). At baseline among men only, the ILI group had lower lumbar spine BMD (g/cm^2^) than the DSE group (*p* = 0.0196), however no other differences between the treatment groups for men were observed. The majority of men and women had BMD in the normal range and very few participants had osteoporosis at baseline. (Table 2).

**Table 1:** Baseline characteristics by gender and randomization arm

Characteristics	Men *N* = 328		*p*-value	Women *N* = 480		*p*-value
	ILI	DSE		ILI	DSE	
Number of Subjects	155	173		235	245	
Age (Years)	60.7 (6.7)	60.1 (6.3)	0.3991	57.1 (6.6)	57.8 (6.6)	0.2653
Race/Ethnicity [N(%)]			0.2643			0.9723
African American	7 (4.5%)	16 (9.3%)		42 (17.9%)	46 (18.8%)	
Caucasian	128 (82.6%)	141 (81.5%)		161 (68.5%)	164 (66.9%)	
Hispanic	8 (5.2%)	8 (4.6%)		18 (7.7%)	21 (8.6%)	
Other	12 (7.7%)	8 (4.6%)		14 (6.0%)	14 (5.7%)	
Body Mass Index (kg/m^2)	33.8 (4.5)	33.4 (4.0)	0.4843	36.1 (5.7)	36.7 (5.5)	0.2346
Smoking Status [N(%)]			0.5381			0.2672
Current	9 (5.8%)	6 (3.5%)		11 (4.7%)	5 (2.1%)	
Former	81 (52.3%)	97 (56.1%)		77 (32.8%)	85 (34.8%)	
Never	65 (41.9%)	70 (40.5%)		147 (62.6%)	154 (63.1%)	
Physical Activity (Maximal MET value)	8.4 (2.1)	8.5 (2.1)	0.5149	7.2 (1.7)	6.9 (1.6)	0.0286
Self-Reported Duration of Diabetes (years)	7.6 (6.7)	7.2 (6.1)	0.6155	5.3 (5.0)	6.0 (5.7)	0.1391
Medications [N(%)]						
Bone Positive*	4 (2.6%)	7 (4.1%)	0.4617	90 (38.3%)	97 (39.6%)	0.7714
Bone Negative**	64 (41.3%)	61 (35.3%)	0.2616	110 (46.8%)	124 (50.6%)	0.4046
TZDs	41 (26.5%)	46 (26.6%)	0.9775	37 (15.7%)	55 (22.6%)	0.0561
Insulin	25 (16.1%)	16 (9.3%)	0.0600	36 (15.3%)	27 (11.2%)	0.1795
eGFR	88.0 (20.2)	89.9 (17.7)	0.3391	94.2 (23.1)	95.8 (24.1)	0.4647
HbA1c	7.17 (1.19)	7.14 (1.13)	0.8419	7.02 (1.00)	7.26 (1.16)	0.0187

*Bone positive medications include calcium, vitamin d, iron, multivitamins, bisphosphonates, sex hormones

**Bone negative medications include antidepressants, thyroid hormones, loop diuretics, corticosteroids, and TZDs

**Table 2:** Baseline outcomes by gender and randomization arm

Characteristics	Men *N* = 328		*p*-value	Women *N* = 480		*p*-value
	ILI	DSE		ILI	DSE	
Total Hip BMD (g/cm^2^)	1.07 (0.12)	1.09 (0.14)	0.2966	1.04 (0.14)	1.04 (0.14)	0.7855
Femoral Neck BMD (g/cm^2^)	0.86 (0.12)	0.88 (0.12)	0.2839	0.87 (0.14)	0.88 (0.14)	0.5717
Lumbar Spine BMD (g/cm^2^)	1.13 (0.15)	1.17 (0.18)	0.0196	1.10 (0.17)	1.12 (0.16)	0.2100
Whole Body BMD (g/cm^2^)	1.18 (0.10)	1.19 (0.12)	0.3145	1.13 (0.12)	1.14 (0.12)	0.7056
Total Hip BMD T-score	0.81 (1.04)	1.05 (1.23)	0.0635	0.27 (1.23)	0.26 (1.33)	0.9800
Lumbar Spine BMD T-score	1.21 (1.54)	1.60 (1.93)	0.0420	0.38 (1.66)	0.47 (1.49)	0.5112
Total Hip BMD*			0.8734			0.1135
Normal range	148 (96.7%)	161 (96.4%)		199 (86.2%)	194 (80.8%)	
Low bone density	5 (3.3%)	6 (3.6%)		29 (12.6%)	45 (18.8%)	
Osteoporosis	0 (0.0%)	0 (0.0%)		3 (1.3%)	1 (0.4%)	
Lumbar Spine BMD*			0.7727			0.6038
Normal range	141 (92.8%)	159 (91.9%)		188 (80.0%)	202 (82.5%)	
Low bone density	11 (7.2%)	14 (8.1%)		43 (18.3%)	41 (16.7%)	
Osteoporosis	0 (0.0%)	0 (0.0%)		4 (1.7%)	2 (0.8%)	

*Low bone density is defined as having a BMD T-score between -2.5 and -1.0; Osteoporosis is defined as having a BMD T-score < -2.5

Weight loss in the ILI was largest at year 1 (8.6% in the ILI vs. 0.7% in the DSE) but remained significantly greater in ILI throughout the trial [12]. When the study intervention ended (median 9.6 years of follow-up), the mean weight loss from baseline was 6.0% in ILI and 3.5% in DSE [13]. The difference in weight loss persisted even at the year 12–16 visit (5.6% in ILI vs 3.6% in DSE; *p* = 0.0059). Physical fitness improvement in the ILI was greatest at year 1 but remained significant through year 4 (last time point measured) compared to the DSE group [14].

At year 8 in adjusted models, percent BMD loss (g/cm^2^) was greater in the ILI group compared with the DSE group for the femoral neck (-4.8 ILI versus -3.5 DSE; *p* = 0.0122) (Table 3). During the year 12–16 visit, BMD loss (g/cm^2^) from baseline was greater in the ILI group compared with the DSE group for the whole body (-0.77 ILI versus 0.71 DSE; p < 0.0029), however there were no differences in the total hip BMD, femoral neck BMD and lumbar spine BMD by intervention group assignment. Further, we repeated this analysis adding time varying HbA1c as a potential confounder and our results were essentially unchanged (data not shown).

**Table 3:** Adjusted* mean (SE) percent change from baseline to follow-up time by randomization arm, overall and stratified by gender

Outcome (% Change from Baseline)		Year 8			LA extension			Yr 8 to LA-E ILI vs DSE
		ILI	DSE	*p*-value	ILI	DSE	*p*-value	*p*-value
Overall	Total Hip BMD	-4.3 (0.36)	-3.7 (0.35)	0.1538	-6.3 (0.42)	-5.4 (0.41)	0.0600	0.4724
	Femoral Neck BMD	-4.8 (0.46)	-3.5 (0.45)	0.0122	-6.8 (0.54)	-6.5 (0.54)	0.6003	0.2106
	Lumbar Spine BMD	1.9 (0.41)	1.4 (0.40)	0.2423	4.0 (0.47)	4.4 (0.46)	0.4595	0.1186
	Whole Body BMD	0.77 (0.36)	1.3 (0.35)	0.1861	-0.77 (0.42)	0.71 (0.42)	0.0029	0.0788
Men	Total Hip BMD	-2.7 (0.69)	-1.6 (0.67)	0.0263	-2.5 (0.75)	-0.73 (0.73)	0.0062	0.3471
	Femoral Neck BMD	-3.5 (1.08)	-2.1 (1.04)	0.0951	-3.0 (1.19)	-3.1 (1.16)	0.9459	0.2494
	Lumbar Spine BMD	5.3 (0.98)	5.1 (0.94)	0.7603	9.8 (1.04)	10.1 (1.00)	0.7040	0.5421
	Whole Body BMD	2.7 (0.76)	3.4 (0.73)	0.2029	1.7 (0.83)	4.6 (0.80)	< .0001	0.0060
Women	Total Hip BMD	-6.1 (0.46)	-6.0 (0.45)	0.8034	-9.5 (0.53)	-9.4 (0.54)	0.7796	0.9359
	Femoral Neck BMD	-6.5 (0.55)	-5.4 (0.54)	0.0735	-10.2 (0.64)	-9.9 (0.65)	0.6377	0.3704
	Lumbar Spine BMD	-0.89 (0.48)	-1.7 (0.48)	0.1237	-0.28 (0.56)	-0.23 (0.56)	0.9409	0.2162
	Whole Body BMD	-0.85 (0.45)	-0.60 (0.45)	0.6311	-2.7 (0.53)	-2.5 (0.54)	0.7186	0.9887

*Adjusted for repeated measures, age, race/ethnicity, study site, baseline total mass, taking bone-positive

medications at baseline, taking bone-negative medications at baseline, and the baseline value of the outcome

Overall models also adjust for gender

In men only, percent BMD loss (g/cm^2^) was greater in the ILI group compared with the DSE group for total hip at both the year 8 and year 12–16 visits (year 8 visit -2.7% ILI versus -1.6% DSE, *p* = 0.0263; and year 12–16 visit -2.5% ILI versus -0.73% DSE, *p* = 0.0062) (Table 3). In women at the year 8 visit, percent BMD loss (g/cm^2^) at the femoral neck was numerically greater in the ILI group compared with the DSE group but the difference did not reach statistical significance (-6.5% ILI versus -5.4% DSE; *p* = 0.0735). This difference was not seen at the year 12–16 visit.

**Fig. 2:**
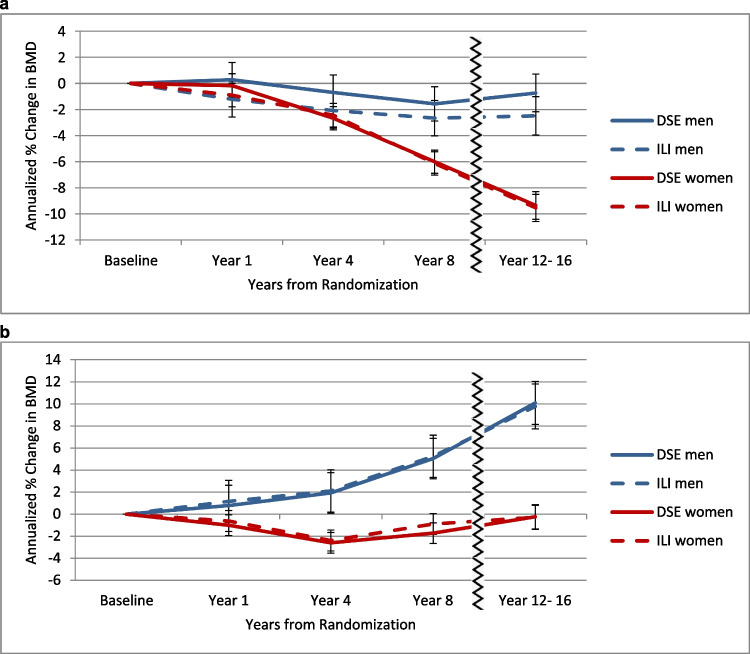
**a** Adjusted* percent change in total hip BMD by randomization arm, stratified by gender. **b**. Adjusted* percent change in spine BMD by randomization arm, stratified by gender. *Analyses adjusted for repeated measures, age, race/ethnicity, study site, baseline total mass, taking bone-positive medications at baseline, taking bone-negative medications at baseline, and baseline hip BMD. The wavy line in Figs. 2a and 2b represents the beginning of the Look AHEAD Extension study

Gender differences in bone loss over time are seen in Fig. 2a and b. Women in both the ILI and DSE groups lost a higher percentage of total hip BMD than men throughout the LA study (mean difference from baseline to year 12–16 in women and men: -9.5% versus -1.1% respectively; p < 0.0001). In men, total hip BMD in the ILI group was lower than in the DSE group over time (*p* = 0.0263 at year 8) and that difference persisted to year 12–16 but was no longer statistically significant (*p* = 0.0600). In women, the ILI and DSE group had similar declines in total hip BMD at year 8 and year 12–16. In contrast, an increase in spine BMD was seen over time in both men and women, with men having a much more pronounced increase in spine BMD over time + 9.1% men v. + 0.21% women (p < 0.0001). However, there was no statistical difference between ILI and DSE groups in spine BMD (see Table 3 and Fig. 2).

At the year 12–16 visit, 6.8%-13.1% of men in both groups experienced > 10% loss in total hip BMD. For women, the corresponding loss was 47.2–48.1% experiencing > 10% loss in total hip BMD. At the spine, 0–2.4% of men experienced > 10% loss of BMD. The corresponding figure for women at the spine was 9.3–12.8%.

There was little usage of bone positive agents in men at the baseline visit (3.4%), however there was a steady increase over the course of the LA study up to 11.3% of men at year 8 and 32.2% by the year 12–16 visit. In women, 39.0% used bone positive agents at baseline, with a decline in usage over the course of the LA study down to 12.7% of women at year 8 and 18.0% by the year 12–16 visit. Similar patterns of bone positive medication use are seen in both treatment arms of the trial.

**Fig. 3:**
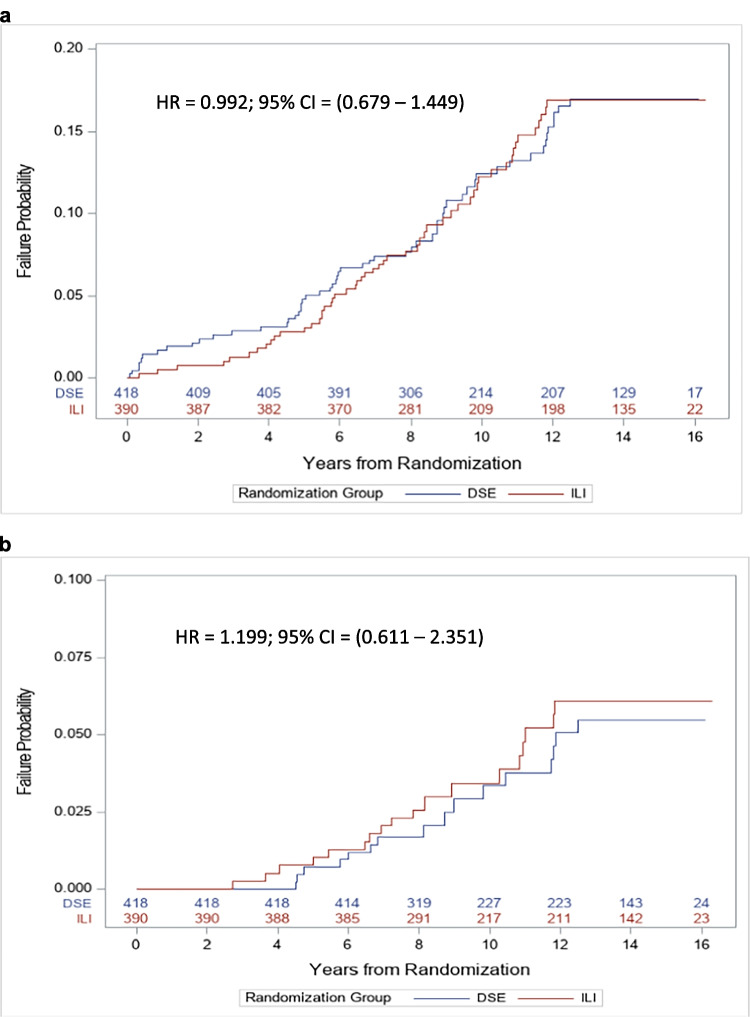
**a** Survival analysis of total fractures within DXA cohort. **b** Survival analysis of frailty fractures within DXA cohort

In a sensitivity analysis of fracture risk in the DXA subgroup, neither incident total fracture (HR = 0.992; 95% CI 0.679 – 1.449; *p* = 0.9664) nor frailty fracture (1.199; 95% CI = (0.611 – 2.351); *p* = 0.5979) were statistically different between the ILI and DSE groups in this subset of Look AHEAD participants with DXA measurements (see Fig. 3a and b). However, among men, the frailty fracture hazard ratio (HR = 1.391; 95% CI = (0.373 – 5.180); *p* = 0.6229) comparing ILI to DSE, while not statistically significant, was similar to the frailty fracture risk estimate for the whole Look AHEAD group with a much larger sample size (*n* = 5145; HR 1.39; 95% CI, 1.02 to 1.89) which was statistically significant. No difference in total fracture risk (HR = 1.040; 95% CI = (0.680 – 1.591); *p* = 0.8579) or frailty fracture risk (HR = 1.118; 95% CI = (0.510 – 2.450); *p* = 0.7809) between ILI and DSE in women was seen.

## Discussion

In the Look AHEAD clinical trial in persons with overweight and obesity and with DM, we previously found that an intensive lifestyle intervention that resulted in intentional long term weight loss and improved fitness increased the risk of frailty fracture [5]. However, the effect of the intervention on bone loss beyond four years was unknown. This current analysis demonstrates that men in the intervention group experienced greater bone loss at the total hip for over a decade. In women, greater bone loss in the intervention group was also observed after one year but the difference between the treatment groups was not sustained long term [3]. We believe the sustained BMD loss observed in the ILI group especially in men helps explain our Look AHEAD findings of increased risk of frailty fractures with long term intentional weight loss. Taken together these long term Look AHEAD findings suggest that when intentional weight loss is planned, consideration of bone preservation and fracture prevention strategies are warranted [15–17]. Additional strategies that merit consideration to reduce risk of falls and fracture during weight loss include incorporation of balance and core strengthening [18].

Our finding of increased bone loss with weight loss is consistent with other reports from the Osteoporotic Fractures in Men Study which found that weight loss regardless of intentionality is associated with an increase in bone loss [19]. The Look AHEAD results are also consistent with prospective studies and short term randomized trials in women that show weight loss whether intentional or unintentional increases risk for bone loss [20–23]. Previous research has shown that BMD loss is greater in women than in men over time [24, 25]. A phenomenon observed in the Look AHEAD data in both the ILI and DSE groups. Our data are also consistent with previous research that has shown that women have higher rates of osteoporosis and greater incidence of fracture than men [26]. Bone loss resulting from intentional weight loss is particularly concerning because persons with Type 2 diabetes, who are often asked to lose weight as a therapeutic strategy of diabetes management, are already at increased risk for fractures compared to individuals without diabetes [27–31]. Moreover, the fracture risk assessment tool (FRAX) underestimates fracture risk in patients with diabetes [32]. Thus, weight loss recommendations for persons with diabetes to improve their glycemic status may increase fracture risk that is underestimated by conventional risk calculators.

We did not observe any differences in bone density changes at the lumbar spine, comparing the two treatment groups. Interestingly, men in both groups experienced increased BMD of the lumbar spine a finding that is consistent with reports from other studies of increasing spine BMD as measured by DXA with aging [33]. These changes observed in the lumbar spine over time have been thought to be related to the development of osteophytes and other degenerative changes that occur with aging particularly in persons over age 60, which can affect the measurement of lumbar BMD by DXA and thus obscure loss of bone that may be occurring at this skeletal site [34–36]. BMD measured by quantitative computed tomography (QCT) is thought to be superior to DXA at detecting bone loss in persons with osteoarthritic changes of the lumbar spine, but this assessment was not done in Look AHEAD [36].

The total number of bariatric surgeries in the DXA subgroup was very small and thus we were not able to examine this surgical procedure’s effect on bone by treatment assignment in the current paper. Although beyond the scope of this paper, it is worth noting that bariatric surgery is an increasingly utilized weight loss strategy in this population [37]. Future work should aim to characterize the long-term implications of surgical weight loss on bone loss, as it is likely augmented due to the greater rate/magnitude of achievable weight loss and increased risk of malabsorptive issues following surgical versus lifestyle intervention [38, 39].

The strengths of the Look AHEAD clinical trial include randomization to group assignment, high levels of retention during longitudinal follow-up, a racially and geographically diverse participant population, and successfully producing long-term intentional weight loss and improvements in physical activity and fitness. Further, the DXA measurement in the DXA substudy and fracture ascertainment in the whole Look AHEAD group were preplanned secondary outcomes of the trial. The DXA data was also rigorously monitored by the DXA quality assurance center and longitudinal performance was assessed with regular scanning of spine and whole body phantoms on each densitometer.

There are several considerations to put interpretation of our findings in context. Look AHEAD did not collect information regarding calcium and vitamin D intake in the DXA subgroup, thus we are unable to examine the observed bone changes in relation to these variables that are known to be important for bone health in both men and women [15, 40, 41]. Additionally, there is evidence to suggest that bone loss with intentional weight loss may be mitigated by calcium supplementation, but this was not the focus of the current study [42]. Adequate calcium and vitamin D intake may be particularly important for persons who plan to engage in intentional weight loss. The Institute of Medicine report on calcium and vitamin D intake currently recommends that persons in the age range of LA participants get 1200 mg/d of calcium and 600–800 IU/d of vitamin D for optimum bone health [43]. It is unknown if Look AHEAD participants reached these recommended levels for calcium and vitamin D intake. Look AHEAD also did not measure serum vitamin D or bone turnover markers in the main trial, but an ancillary study has recently been funded by the NIH that will measure these bone biomarkers that will allow us to examine metabolomic pathways that may help explain our findings. In addition, the effect of specific exercise routines that emphasize balance and improving core strength to reduce risk of falls and fractures warrants further study.

Another limitation of the substudy is use of a DXA scanner in the setting of weight change. With obesity, the long-term precision of the DXA scanner is reduced which may result in attenuation of associations between changes in weight and BMD [44]. While soft tissue results were corrected in Look AHEAD to account for underestimation of fat mass at the DXA quality assurance center, the reduced precision may have affected our results [10]. Further, these results may not be generalizable to all those with type 2 diabetes because Look AHEAD was not able to perform a DXA scan on persons weighing more than 300 lbs due to the weight limit of the densitometer and Look AHEAD participants were healthy volunteers for a long-term clinical trial. Caution is also warranted for generalizing these results to persons without diabetes. The sample size of the DXA substudy was much smaller (*n* = 808) compared to the entire Look AHEAD study (*n* = 5143), thus power to detect differences in fracture outcomes is much reduced. We acknowledge that multiple statistical comparisons have been made increasing the risk of making a Type I error. Further, we acknowledge that the weight loss differences between the ILI and DSE attenuated over time and that unintentional weight loss may have occurred in both ILI and DSE as the study group aged. Another limitation is the absence of any data on possible mechanism(s) for the observed differences and additional study appears warranted to understand these findings from a mechanistic point of view.

## Conclusion

Long term intentional weight loss was associated with greater bone loss at the hip in men long term and in women in the shorter term. These results taken with the previously published Look AHEAD fracture data from the entire clinical trial showing increased frailty fracture risk in the ILI group with intentional weight loss suggest that when intentional weight loss is planned, consideration of bone density preservation and fracture prevention strategies is warranted.

### Supplementary Information

Below is the link to the electronic supplementary material.

Supplementary file1 (DOCX 19 KB)

## Data Availability

Data that support the findings from the Look AHEAD trial (Action for Health in Diabetes) can be accessed through application to the National Institute of Diabetes and Digestive and Kidney Diseases; NIDDK Central Repository: https://repository.niddk.nih.gov/studies/look-ahead/.
