# Supplementary material for: The long-term effect of intentional weight loss on changes in bone mineral density in persons with type 2 diabetes: results from the Look AHEAD randomized trial
Source: Arch Osteoporos. 2023 Jul 14;18(1):97. doi: 10.1007/s11657-023-01303-0 (PMC10348976; doi:10.1007/s11657-023-01303-0)
Supplement: Supplementary file 1 — Supplementary file1 (DOCX 19 KB) [file 11657_2023_1303_MOESM1_ESM.docx]

**Appendix: Look AHEAD Extension Study Group**

**Clinical Sites**

The Johns Hopkins University Jeanne M. Clark, MD, MPH^1^; Lee Swartz^2^; Dawn Jiggetts^2^; Jeanne Charleston, RN^3^; Lawrence Cheskin, MD^3^; Nisa M. Maruthur, MD, MHS ^3^; Scott J. Pilla, MD, MHS^3^; Danielle Diggins; Mia Johnson

Pennington Biomedical Research Center George A. Bray, MD^1^; Frank L. Greenway, MD^1^; Donna H. Ryan, MD^3^; Catherine Champagne, PhD, RD^3^; Valerie Myers, PhD^3^; Jeffrey Keller, PhD^3^; Tiffany Stewart, PhD^3^; Jennifer Arceneaux, RN^2^; Karen Boley, RD, LDN^2^; Greta Fry, LPN; Lisa Jones; Kim Landry; Melissa Lingle; Marisa Smith^2^.

The University of Alabama at Birmingham Cora E. Lewis, MD, MSPH^1^; Sheikilya Thomas, PhD, MPH^2^; Stephen Glasser, MD^3^; Gareth Dutton, PhD^3^; Amy Dobelstein; Sara Hannum; Anne Hubbell, MS; DeLavallade Lee; Phyllis Millhouse, L. Christie Oden; Cathy Roche, PhD, RN, BSN; Jackie Grant; Janet Turman

Harvard Center

*Massachusetts General Hospital*. David M. Nathan, MD^1^; Valerie Goldman, MS, RDN^2^; Linda Delahanty, MS, RDN^3^;

Mary Larkin, MS, RN; Kristen Dalton, BS; Roshni Singh, BS; Melanie Ruazol, BS

*Joslin Diabetes Center*: Joslin Diabetes Center: Medha N Munshi, MD^1^; Sharon D. Jackson, CCRC, MS, RD, CDE^2^; Roeland J.W. Middelbeek MD^3^; A. Enrique Caballero, MD, Anthony Rodriguez

*Beth Israel Deaconess Medical Center*: George Blackburn, MD, PhD^1*^; Christos Mantzoros, MD, DSc^3^; Ann McNamara, RN

University of Colorado Anschutz Medical Campus Holly Wyatt, MD^1^; James O. Hill, PhD^1^; Jeanne Anne Breen, MS^2^; Marsha Miller, MS, RD^2^; Debbie Bochert; Suzette Bossart; Paulette Cohrs, RN, BSN; Susan Green; April Hamilton, BS, CCRC; Eugene Leshchinskiy; Loretta Rome, TRS.

Baylor College of Medicine John P. Foreyt, PhD^1^; ; Molly Gee, MEd, RD^2^; Henry Pownall, PhD^3^; Ashok Balasubramanyam, MBBS^3^; Chu-Huang Chen, MD, PhD^3^; Peter Jones, MD^3^; Michele Burrington, RD, RN; Allyson Clark Gardner, MS, RD; Sharon Griggs; Michelle Hamilton; Veronica Holley; Sarah Lee; Sarah Lane Liscum, RN, MPH; Susan Cantu-Lumbreras; Julieta Palencia, RN; Jennifer Schmidt; Jayne Thomas, RD; Carolyn White; Charlyne Wright, RN; Monica Alvarez, PCT

The University of Tennessee Health Science Center

*University of Tennessee East.* Karen C. Johnson, MD, MPH^1^; Beate Griffin, RN, BS^2^; Mace Coday, PhD^3^; Donna Valenski, Lisa Jones; Karen Johnson, RN

*University of Tennessee Downtown.* Karen C. Johnson, MD, MPH^1^; Beate Griffin, RN, BS^2^; Helmut Steinburg, MD^3^

University of Minnesota Robert W. Jeffery, PhD^1^; Tricia Skarphol, MA^2^; John P. Bantle, MD^3^; J. Bruce Redmon, MD^3^; Kerrin Brelje, MPH, RD; Carolyne Campbell; Mary Ann Forseth, BA; Soni Uccellini, BS; Mary Susan Voeller, BA

Columbia University Medical Center  Blandine Laferrère, MD, PhD^1^; Xavier Pi-Sunyer, MD^1^; Jennifer Patricio, MS^2^; Jose Luchsinger, MD^1^; Priya Palta, PhD,MHS^3^; Jennifer Patricio, MS^2^; Sarah Lyon, Kim Kelly

University of Pennsylvania Thomas A. Wadden, PhD^1^; Barbara J. Maschak-Carey, MSN, CDE^2^; Robert I. Berkowitz, MD^3^; Ariana Chao, PhD, CRNP^3^; Renee Davenport; Katherine Gruber, CRNP; Sharon Leonard, RD; Olivia Walsh, BA

University of Pittsburgh  John M. Jakicic, PhD^1^; Jacqueline Wesche-Thobaben, RN, BSN, CDE^2^; Lin Ewing, PhD, RN^3^; Andrea Hergenroeder, PhD, PT, CCS^3^; Mary Korytkowski, MD^3^; Susan Copelli, BS, CTR; Rebecca Danchenko, BS; Diane Ives, MPH; Juliet Mancino, MS, RD, CDE, LDN; Lisa Martich, BS, RD, LDN; Meghan McGuire, MS; Tracey Y. Murray, BS; Linda Semler, MS, RD, LDN; Kathy Williams, RN, MHA

The Miriam Hospital/Brown Medical School  Rena R. Wing, PhD^1^; Caitlin Egan, MS^2^; Elissa Jelalian, PhD^3^; Jeanne McCaffery, PhD^3^ ; Kathryn Demos McDermott, PhD^3^; Jessica Unick, PhD^3^; Kirsten Annis, BA; Jose DaCruz; Ariana Rafanelli, BA

The University of Texas Health Science Center at San Antonio Helen P. Hazuda, PhD^1^; Juan Carlos Isaac, CCRC, BSN^2^; Prepedigna Hernandez, RN

VA Puget Sound Health Care System / University of Washington Steven E. Kahn, MB, ChB^1^; Edward J. Boyko, MD, MPH^3^; Elaine Tsai, MD^3^; Lorena fWright, MD^3^; Karen Atkinson, RN, BSN^2^; Ivy Morgan-Taggart; Jolanta Socha, BS; Heidi Urquhart, RN

Southwestern American Indian Center, Phoenix, Arizona and Shiprock, New Mexico William C. Knowler, MD, DrPH^1^; Paula Bolin, RN, MC^2^; Harelda Anderson, LMSW^2^, Sara Michaels, MD^3^; Ruby Johnson; Patricia Poorthunder; Janelia Smiley

University of Southern California Anne L. Peters, MD^1^; Siran Ghazarian, MD^2^; Elizabeth Beale, MD^3^; Edgar Ramirez; Gabriela Rodriguez, MA; Valerie Ruelas MSW, LCSW; Sara Serafin-Dokhan; Martha Walker, RD; Marina Perez

**Coordinating Center**

## Wake Forest University  Mark A. Espeland, PhD^1^; Lynne E. Wagenknecht, DrPH^1^; Judy L. Bahnson, BA, CCRP^3^; David Reboussin, PhD^3^; Mike E. Miller, PhD^3^; Peter Brubaker, PhD^3^; Nicholas Pajewski, PhD^3^; Michael Bancks, PhD^3^; Jingzhong Ding, PhD^3^; Gagan Deep, PhD^3^; Kathleen Hayden, PhD^3^; Stephen R. Rapp, PhD^3^; Felicia Simpson, PhD^3^; Haiying Chen, PhD, MM^3^; Bonnie C. Sachs, PhD^3^; Denise Houston, PhD^3^; Shyh-Huei Chen, PhD^3^; Andrea Anderson, MS; Jerry M. Barnes, MA; Mary Barr; Tara D. Beckner; Delilah R. Cook; Carrie C. Williams, MA, CCRP; Joni Evans, MS; Katie Garcia, MS; Sarah A. Gaussoin, MS; Carol Kittel, MS; Lea Harvin, BS; Marjorie Howard, MS; Joni Evans, MS; James Lovato, MS; Rebecca H. Neiberg, MS; June Pierce, AB; Debbie Steinberg, BS; Christopher Webb; Jennifer Walker, MS; Michael P. Walkup, MS; Carolyn Watkins

**Central Resources Centers**

Central Laboratory, Northwest Lipid Metabolism and Diabetes Research Laboratories Santica M. Marcovina, PhD, ScD^1^; Jessica Hurting^2^; John J. Albers, PhD^3^, Vinod Gaur, PhD^4^

## DXA Reading Center, University of California at San Francisco Michael Nevitt, PhD^1^; Ann Schwartz, PhD^2^; John Shepherd, PhD^3^; Michaela Rahorst; Lisa Palermo, MS, MA; Susan Ewing, MS; Cynthia Hayashi; Jason Maeda, MPH

**Federal Sponsors**

National Institute of Diabetes and Digestive and Kidney Diseases Mary Evans, PhD; Van S. Hubbard, MD, PhD; Susan Z. Yanovski, MD

Centers for Disease Control and Prevention Edward W. Gregg, PhD; Ping Zhang, PhD

**Funding and Support**           Funded by the National Institutes of Health through cooperative agreements with the National Institute on Aging: AG058571 and National Institute of Diabetes and Digestive and Kidney Diseases: DK57136, DK57149, DK56990, DK57177, DK57171, DK57151, DK57182, DK57131, DK57002, DK57078, DK57154, DK57178, DK57219, DK57008, DK57135, and DK56992. Additional funding was provided by the National Heart, Lung, and Blood Institute; National Institute of Nursing Research; National Center on Minority Health and Health Disparities; NIH Office of Research on Women’s Health; and the Centers for Disease Control and Prevention. This research was supported in part by the Intramural Research Program of the National Institute of Diabetes and Digestive and Kidney Diseases. The Indian Health Service (I.H.S.) provided personnel, medical oversight, and use of facilities. The opinions expressed in this paper are those of the authors and do not necessarily reflect the views of the I.H.S. or other funding sources.

Additional support was received from The Johns Hopkins Medical Institutions Bayview General Clinical Research Center (M01RR02719); the Massachusetts General Hospital Mallinckrodt General Clinical Research Center and the Massachusetts Institute of Technology General Clinical Research Center (M01RR01066); the Harvard Clinical and Translational Science Center (RR025758-04); the University of Colorado Health Sciences Center General Clinical Research Center (M01RR00051) and Clinical Nutrition Research Unit (P30 DK48520); the University of Tennessee at Memphis General Clinical Research Center (M01RR0021140); the University of Pittsburgh General Clinical Research Center (GCRC) (M01RR000056), the Clinical Translational Research Center (CTRC) funded by the Clinical & Translational Science Award (UL1 RR 024153) and NIH grant (DK 046204); the VA Puget Sound Health Care System Medical Research Service, Department of Veterans Affairs; and the Frederic C. Bartter General Clinical Research Center (M01RR01346).

The following organizations have made major contributions to Look AHEAD: FedEx Corporation; Health Management Resources; LifeScan, Inc., a Johnson & Johnson Company; OPTIFAST® of Nestle HealthCare Nutrition, Inc.; Hoffmann-La Roche Inc.; Abbott Nutrition; and Slim-Fast Brand of Unilever North America.

Some of the information contained herein was derived from data provided by the Bureau of Vital Statistics, New York City Department of Health and Mental Hygiene.

______________________________

^1^ Principal Investigator

^2^ Program Coordinator

^3^ Co-Investigator

*Deceased

All other Look AHEAD staffs are listed alphabetically by site.
